# The remarkable activity and stability of a highly dispersive *beta*-brass Cu-Zn catalyst for the production of ethylene glycol

**DOI:** 10.1038/srep20527

**Published:** 2016-02-09

**Authors:** Molly Meng-Jung Li, Jianwei Zheng, Jin Qu, Fenglin Liao, Elizabeth Raine, Winson C. H. Kuo, Shei Sia Su, Pang Po, Youzhu Yuan, Shik Chi Edman Tsang

**Affiliations:** 1 Wolfson Catalysis Centre, Department of Chemistry, University of Oxford, Oxford, OX1 3QR, UK; 2 State Key Laboratory of Physical Chemistry of Solid Surfaces and National Engineering Laboratory for Green Chemical Production of Alcohols-Ethers-Esters, Collaborative Innovation Center of Chemistry for Energy Materials, College of Chemistry and Chemical Engineering, Xiamen University, Xiamen 361005, PR China; 3 Metallurgy and Materials, University of Birmingham, B15 2TT, UK

## Abstract

Incorporation of Zn atoms into a nanosize Cu lattice is known to alter the electronic properties of Cu, improving catalytic performance in a number of industrially important reactions. However the structural influence of Zn on the Cu phase is not well studied. Here, we show that Cu nano-clusters modified with increasing concentration of Zn, derived from ZnO support doped with Ga^3+^, can dramatically enhance their stability against metal sintering. As a result, the hydrogenation of dimethyl oxalate (DMO) to ethylene glycol, an important reaction well known for deactivation from copper nanoparticle sintering, can show greatly enhanced activity and stability with the CuZn alloy catalysts due to no noticeable sintering. HRTEM, nano-diffraction and EXAFS characterization reveal the presence of a small *beta*-brass CuZn alloy phase (body-centred cubic, *bcc*) which appears to greatly stabilise Cu atoms from aggregation in accelerated deactivation tests. DFT calculations also indicate that the small *bcc* CuZn phase is more stable against Cu adatom migration than the *fcc* CuZn phase with the ability to maintain a higher Cu dispersion on its surface.

Cu-based catalysts are widely used for many important reactions in the chemical industry, including methanol synthesis^1^,^2^,^3^, the water-gas shift reaction^4^,^5^,^6^, steam reforming^7^,^8^,^9^ and hydrogenation of dimethyl oxalate (DMO) to ethylene glycol (EG)^10^,^11^,^12^,^13^,^14^,^15^,^16^,^17^. The syngas (CO/H_2_) conversion to methanol over Cu/ZnO/Al_2_O_3_ is perhaps one of the most studied catalytic reactions over the past 40 years^18^. Electronic and structural modifications of the Cu phase, by varying support and additive(s), for enhanced catalytic performance have been investigated^1^,^2^,^19^. However a detailed understanding of this promotional effect is not yet reported due to the difficulties of detailed structural analysis of small alloy clusters on a multi-component support. Thanks to the rapid improvement of analytical surface science characterization techniques and theoretical methodologies, the potential effects induced by supported alloy catalysts are becoming clearer. Using advanced instruments researchers have recently shown that a very small number of Zn atoms are reduced from the ZnO support. These Zn atoms can decorate the Cu nanoparticle leading to a subtle change in electronic structure^20^,^21^,^22^. As a result, the intermediate formate ion has greater stabilization than that on the unmodified Cu surface^20^,^21^,^22^. Further reports have demonstrated that the decoration of stepped copper particles by Zn atoms in the industrial Cu/ZnO/Al_2_O_3_ catalyst is also identified, accounting for high activity and selectivity^2^. It has also recently been reported that addition of Ga^3+^ into Cu-ZnO precursors by co-precipitation gives a catalyst with exceptionally small but stable Cu crystallites (~0.5–2 nm) under methanol synthesis conditions^23^,^24^,^25^. One possibility is that the presence of Ga^3+^ facilitates the reduction of Zn atoms from ZnO, stabilizing the small Cu particles. It is long believed that Zn atoms are essential in catalysts for textural dispersion of the copper phase^18^,^19^,^20^,^26^,^27^,^28^,^29^, but the precise interaction between Zn and Cu still remains unclear. Moreover, the mechanism of stabilization from Zn and the configuration between Zn and Cu also need to be investigated.

In this work, we have systematically investigated Ga^3+^ doping into Cu-ZnO by controlling chemical composition and calcination temperature. We were particularly interested to develop these composite materials as effective catalysts for the hydrogenation of DMO to EG reaction due to the recent incentive to develop this non-oil based reaction route for chemicals synthesis. EG is an important raw material in the manufacture of polyester fibers and fabrics, and polyethylene terephthalate, widely used in our society^10^. It is produced almost exclusively from ethylene, derived from petroleum cracking, via hydrolysis of the intermediate ethylene oxide. However, the synthesis of ethylene glycol from natural gas, coal or biomass is becoming a strategic process due to the diversification in energy and chemical supplies for many nations. In fact, the insecure long-term prospect of petroleum supplies prompts the development of these non-oil based chemical processes. This involves the formation of syngas from coal or natural gas, followed by the coupling of CO with methyl nitrite to DMO catalysed by Pd and then hydrogenation to EG by Cu^11^,^12^,^13^,^14^,^15^. This synthesis route appears to be especially important for countries like China and the USA where coal is abundant. While the coupling reaction is efficiently taken place, one key issue of this new process is the latter catalytic hydrogenation reaction. This step has inherent problems with poor stability and short lifetime of the copper-based catalyst during the vapour phase hydrogenation of DMO to EG^11^,^12^,^15^,^16^,^17^. There is a strong tendency for copper nanoparticles to grow into larger crystallites through migration and coalescence, hence losing copper surface area during this reaction. Thus, finding a practical way to create and maintain highly dispersed copper on a catalyst surface has been proposed to be an important strategy to improve catalytic performance. Approaches to stabilize a high surface area of Cu comprise alloying Cu with a higher-melting point metal, encapsulation of the Cu nanoparticle in porous templates and the use of metal oxides with strong metal-support interaction to produce small Cu nanoparticles are included. In addition to stabilizing them against coalescence, the metal-support interaction of ZnO and Cr_2_O_3_ have been documented^27^,^28^,^29^,^30^,^31^.

Here, we report the progressive formation of Zn^0^ when Ga^3+^ is added to the Cu/ZnO precursors under H_2_, which gives rise to CuZn nanoclusters. A clear inverse correlation between Zn^0^ concentration and Cu particle size in the catalysts is, for the first time, revealed. From high resolution transmission electron microscopy (HRTEM), nano-diffraction, and extended X-ray absorption fine structure (EXAFS) analyses, a *bcc* CuZn phase can be identified. According to our density functional theory (DFT) calculations the *bcc* CuZn is more stable against metal sintering than the corresponding *fcc* Cu or *fcc* CuZn. Thus, this new type of catalyst shows remarkable activity and stability for the hydrogenation of DMO to EG. This conclusion, although demonstrated for the hydrogenation of DMO reaction, is expected to be applicable to other Cu catalyzed reactions that are susceptible to Cu sintering. It is thus believed that this finding can offer a new way to stabilize surface Cu clusters for high activity and stability.

## Results

### Progressive addition of Ga^3+^

Through incorporation of Ga^3+^ ions into the Cu and Zn containing precursor system a series of Zn modified Cu catalysts have been prepared by co-precipitation with control of precursor injection rate, pH value and precipitation temperature (refer to Methods and supplementary information (SI)). The calcination temperatures of the precursors and the chemical compositions were thoroughly investigated to correlate catalytic performance to various synthesis conditions of these Cu catalysts. Upon heating the solid mixed phases CuO, ZnO, ZnGa_2_O_4_ and (Cu,Zn)_5_(CO_3_)_2_(OH)_16_ were identified by powder XRD (Fig. S1). Under H_2_, the reduction of CuO to Cu occurred at lower temperature when doping with Ga^3+^ (see TPR in Fig. S2). As previously reported, XRD was unable to characterise the small Cu containing nano-clusters produced after reduction^23^,^24^,^25^. Thus, detailed XPS analysis was conducted which detected Cu^0^ (total reduction) and Ga^3+^ (no reduction) exclusively. XPS also indicated partial reduction of Zn^2+^ to form Zn^0^ after the H_2_ treatment of the co-precipitated precursors. Fig. S3a clearly confirms that the Zn 2p signal is composed of two peaks. They can be resolved to be 1023 eV and 1021 eV matching to the binding energies of Zn^2+^ and Zn^0^, respectively. Thus, the surface composition of catalysts analysed by XPS is presented in Table 1. It is apparent that the surface concentration of Zn^0^ varies with chemical composition and calcination temperature. The Zn^0^/(Zn^0^ + Zn^2+^) ratio increased as the content of Ga^3+^ increased to a maximum of 0.32 at a chemical composition of 43%Cu, 52%Zn, and 5%Ga. The ratio then decreased as the Ga^3+^ concentration was increased further. When using the same chemical composition but varying different calcination temperatures, the Zn^0^/(Zn^0^ + Zn^2+^) ratio was found to reach a maximum value when the sample was calcined at 330 °C. The CZG29 sample gave the highest Zn^0^/(Zn^0^ + Zn^2+^) ratio among all the studied catalysts, which means it has the highest ratio of Zn atoms decorated on Cu nanoparticles, and has the highest Zn^0^/Cu ratio than the other samples. Although XPS is a useful technique to obtain near surface composition, it cannot provide the topmost atomic layer analysis because the photoelectron escaping depth can reach to few nano-meters. Therefore, by using low energy ion, the penetration depth to the solid sample can be greatly restricted. As a result, apart from the XPS analysis, we also used the more surface-sensitive technique of high-sensitive low-energy ion scattering (HS-LEIS), which provided the elemental composition of the outermost surface (refer to SI for the details of the experiment of HS-LEIS), to characterise two selected samples, CZG28 and CZG29. As seen from Fig. S3b and insert table, an even higher degree of Zn decoration on the outmost surface of Cu of these two samples than that of XPS is depicted.

Also, in the catalyst synthesis, an addition of Ga^3+^ into the Cu and Zn containing precursor system can clearly create a ZnGa_2_O_4_ phase, which depends on calcination temperature (>330 °C) and composition (Fig. S1). The apparent co-existence of ZnGa_2_O_4_ and ZnO phases (both identified in XRD) suggests the formation of a material interface between these two phases. It has been reported that the formation of heterojunction interface with two semiconducting solid phases (ZnGa_2_O_4_ and ZnO) with different band energies could lead to charge separation of the phases. In this case, the reducibility of the refractory ZnO support phase is greatly enhanced^32^. From the XPS result, the concentration of Zn^0^ indeed shows an optimum response to the presence of ZnGa_2_O_4_ and ZnO phases.

### DMO hydrogenation

**Figure 1:**
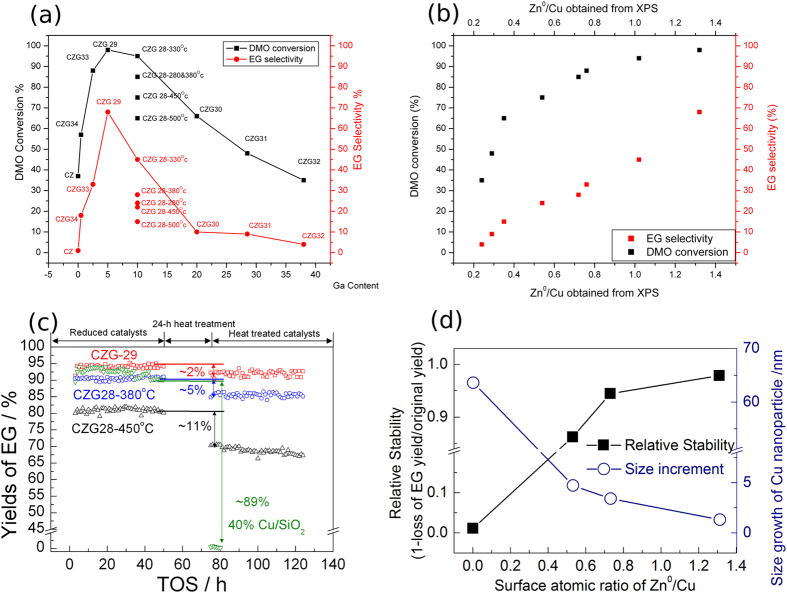
(**a**) Catalytic performances of CZG samples prepared with various chemical compositions and calcination temperatures (all calcined at 330 °C unless otherwise indicated; 330 ^o^C was found to be the optimal temperature for the establishment of ZnGa_2_O_4_/ZnO phases over other phases according to XRD) under reaction conditions of 220 °C, 3.0 MPa, H_2_/DMO = 80, WHSV = 0.6 g g^−1^ h ^−1^ and m_catal_. = 100 mg; (**b**) The correlation between Zn^0^/Cu obtained from XPS result with DMO hydrogenation catalytic performances of CZG samples; (**c**) Comparison of catalytic performance and stabilities of Cu-containing catalysts; reaction conditions: P(H_2_) = 3.0 MPa, H_2_/DMO = 80 (v/v), Temperature = 240 °C, WLSHV = 0.6 g·g^−1^·h^−1^ and m_catal_. = 100 mg (20 mg Cu/SiO_2_ was diluted by 80 mg quartz sand); subjected to heat treatment at 400 °C for 24 h under N_2_ before resuming the testing; (**d**) the correlation of Zn^0^/Cu ratio with stability and particle size change.

It is accepted that hydrogenation of DMO to EG can be effectively catalyzed by a Cu surface^10^,^11^,^12^,^13^,^14^,^15^,^16^,^17^. As a result, this reaction was studied over the series of CuZnGa catalysts. It can be seen from Fig. 1a that as the Ga^3+^ concentration increases, the activity increases, reaching a maximum value at the CZG29 sample. The activity then decreases as the Ga^3+^ concentration further increases. The EG selectivity has a similar trend to the activity. It can be seen that the calcination temperature of the CuZnGa precursors also affects the catalytic performance with a calcination temperature of 330 °C showing the best performance amongst the CZG28 series. Comparing the change in Ga^3+^ concentration with Zn^0^/(Zn^0^ + Zn^2+^) in Table 1 shows a trend that the activity and selectivity follow Zn^0^/(Zn^0^ + Zn^2+^) or Zn^0^/Cu ratio closely, see Fig. 1b. The catalytic performance of catalysts with an *in-situ* treatment (by raising the temperature to 400 °C for 24 h under N_2_) shows very different behaviours than that without the high temperature treatment (Fig. 1c). A highly active Cu/SiO_2_ catalyst containing no Zn was also introduced as a comparison. It can be seen that the EG yield of the Cu/SiO_2_ catalyst with an unmodified Cu surface, where the Cu particle size is 3.1 nm, reaches 95%, with both impressive activity and selectivity in this hydrogenation reaction. This differs from the methanol synthesis reaction where a pure Cu surface does not deliver an acceptable selectivity without Zn inclusion^1^,^2^,^3^,^18^,^20^,^21^,^22^. Thus, the electronic promotion from alloying Cu with Zn is perhaps unnecessary for this reaction, in addition, this catalyst is well-known for deactivation due to inherent problems of poor stability^11^,^12^,^15^,^16^,^17^. The EG yield of the Cu/SiO_2_ catalyst after heat treatment significantly decreased by 89%. In contrast, the CZG29 sample containing Zn^0^/Cu ratio of 1.32 only drops by 2% yield of EG after the 24 h accelerated heat treatment at 400 °C. Interestingly, samples CZG28–380 (Zn^0^/Cu ratio of 0.72) and CZG28-450 (Zn^0^/Cu ratio of 0.54) with intermediate Zn^0^/Cu ratios deactivated by 5 and 11% of EG yields accordingly. Thus, there is a clear correlation of stability with Zn^0^/Cu ratio, which suggests the active Cu particles might have severe sintering after heat treatment but the presence of Zn^0^ minimized the extent of sintering. Fig. S4 shows the TEM images before and after 24 h heat treatment of the samples. It can be seen that after 24 h heat treatment, Cu/SiO_2_ indeed suffered from serious particle aggregation (average diameter increased from 3.1 nm to 66.7 nm), whereas CZG29 maintained the small particle size (Figs S4 and S5). The results shown in Fig. 1d summarize the importance of Zn^0^/Cu which appears to stabilize the Cu nanoparticles from sintering at higher temperature, hence, minimizing the extent of deactivation. CZG29 is shown to be the most active and stable catalyst among those Cu-containing catalysts. Therefore, regarding the favorable Zn solubility into the Cu lattice, it is logical to assume that the Cu clusters from CuZnGa may be stabilized by Zn^0^ atoms through the formation of nano-CuZn alloy.

**Figure 2:**
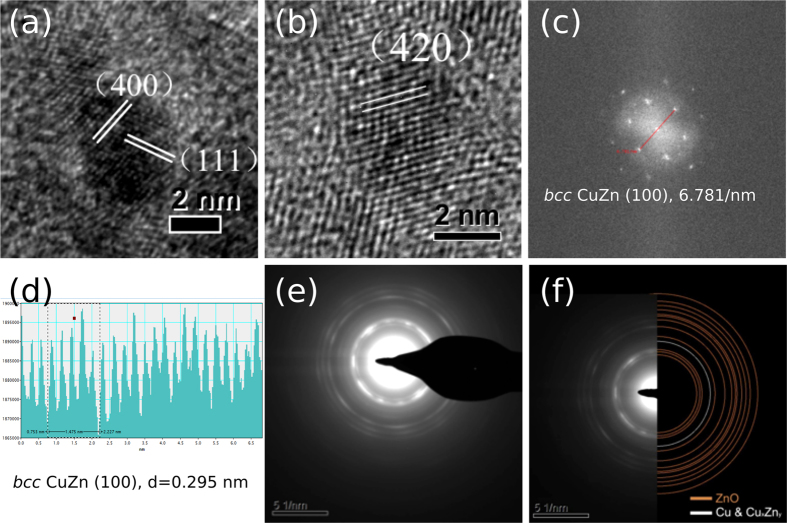
Structural analysis of CZG29 sample: (**a,b**) HRTEM showing (400) and (420) *bcc* CuZn phase surrounding (111) *fcc* Cu nanoparticle core; (**c**) nano-diffraction of selected *bcc* CuZn phase; notice that the less well-defined diffraction spots could be arisen from the small sized and highly strained crystallite; (**d**) (100) phase of d = 0.295 nm; (**e**) selected area of diffraction rings; (**f**) diffraction rings matching well with simulated Braggs’ diffractions of Cu, ZnO and *bcc* CuZn phases.

**Figure 3:**
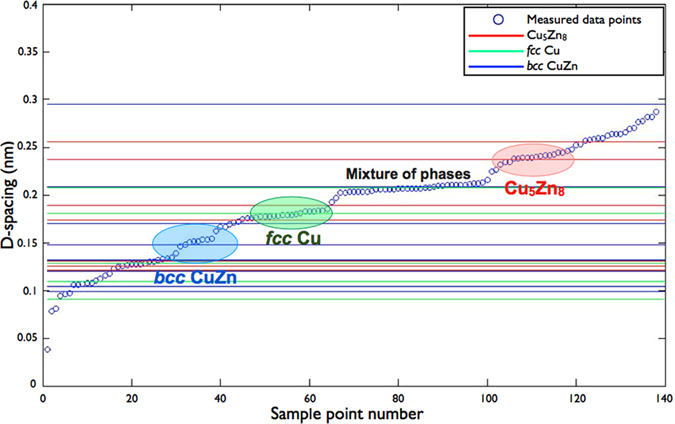
Statistical d-spacing measurements from the lattice fringes and the corresponding FFT of selected HR-STEM images. The coloured lines of Cu_5_Zn_8_ (Red), Cu (Green) and CuZn (Blue) are the d-spacing of each structures: Cu (00–001–1241); CuZn (04–003–4270) and Cu_5_Zn_8_ (00–025–1128).

According to the phase diagram of Cu-Zn^33^, when the concentration of Zn is below 35% the CuZn alloy will stay as the *fcc* structure, as the same as Cu. However, when the Zn concentration is higher than 50%, as shown in most samples in our case, the structure tends to change to *bcc*. Then a change to complex cubic (Cu_5_Zn_8_) is seen when the Zn concentration is higher than 62%, and finally to hcp if Zn ratio is higher than 75%. These structural changes are thought to be attributed to the increasing electron contribution from Zn into anti-bonding bands of CuZn, hence making the primary structures unstable^34^. XRD could not be used to indicate the structure of these small crystallites of below 3–4 nm due to severe peak broadening. Thus, we carried out metal particle mapping and analysis by HRTEM and high resolution scanning transmission electron microscopy (HR-STEM). Close examination of the selected co-precipitated sample (CZG-29), containing the highest Zn^0^/Cu ratio, by microscopy clearly indicates a mixture of phases. The most prevalent phase in this reduced sample is the bulk ZnO (observable by XRD). There is also a clear evidence of the formation of the other phases: *fcc* Cu (may contain Zn as *fcc* CuZn with same lattice parameter), *bcc* CuZn and trace complex cubic Cu_5_Zn_8_ phases (at about 2 nm) (Fig. 2). Although the majority of nano-diffraction rings were generated from the substrate material, ZnO, Fig. 3 shows the statistical analysis of the Cu containing phases from the HR-STEM bright field images, when 140 sampling points were carried out. The coloured lines of Cu_5_Zn_8_ (Red), Cu (Green) and CuZn (Blue) represent the characteristic d-spacings of each structure: Cu (00–001–1241), CuZn (04–003–4270) and Cu_5_Zn_8_ (00–025–1128). The d-spacings of crystals were measured from the lattice fringes and the corresponding fast-Fourier Transform (FFT) images of selected HR-STEM images (Fig. S6). The three selected areas, (blue, green and red) in Fig. 3 indicate the frequent appearance of d-spacings, which is 0.148 nm from *bcc* CuZn, 0.181 nm from *fcc* Cu and 0.237 nm from complex cubic Cu_5_Zn_8_. Thus, nano-diffraction and microscopic techniques give a direct evidence of the presence of *bcc* CuZn and trace complex cubic Cu_5_Zn_8_ phases in the reduced CuZnGa catalyst (CZG29) when there is a large quantity of Zn^0^ in contact with Cu nanoparticles. This was predicted from the Cu-Zn phase diagram. On the other hand, the CZ catalyst without Ga (Cu/ZnO sample) contained exclusively the *fcc* Cu phase.

To further confirm the incorporation of Zn^0^ into the nano Cu lattice, extended X-ray absorption fine structure (EXAFS) of Cu was studied. This technique explores the local structural information of the Cu atom. The experimental data for the reduced CuZnGa samples was recorded and satisfactory R fittings were achieved, as can be seen in Fig. S7, and Table 2. It was found that the spectra of most of the catalysts in present work could not be well modeled using only scattering parameters from metallic *fcc* Cu. Therefore, a mixed-structure model is introduced and it is found that the combination of both *fcc* metallic Cu and *bcc* CuZn alloy structures give significantly improved fits to the experimental spectra. We did not attempt to fit the complex cubic Cu_5_Zn_8_ since there was only a small quantity present in the sample. The EXAFS were fitted using scattering paths of Cu-Cu (2.56 Å) from metallic *fcc* Cu model, Cu-Zn (2.56 Å) and Cu-Cu (2.99 Å) from *bcc* Cu-Zn alloy model. Notice that the longer distance of Cu-Cu (2.99 Å) in the *bcc* Cu-Zn structure is distinctive from the shorter Cu-Cu (2.56 Å) of the *fcc* Cu model and Cu-Zn (2.56 Å) of the *bcc* Cu-Zn model. As shown in Table 2, all R-factors are below 0.8% with the coordination number of the Cu-Zn bond, derived from 2.56 Å scattering path, ranging from 6 to 9. As for the CN(Cu-Cu), derived from 2.99 Å scattering path, it can be seen that the CZG29 sample had the highest coordination number of *bcc* Cu-Cu bond, thus indicates the highest concentration of *bcc* Cu-Zn alloy. We also found the *bcc* CuZn concentration is consistent with the concentration of Zn^0^/Cu observed from the XPS result (Fig. S8). In addition, no Cu-Cu bond of 2.99 Å was observed in the 500 °C-calcined sample which suggests that no *bcc* CuZn alloy was formed during the reduction procedure if the CuZnGa precursor has been calcined at high temperature. The EXAFS confirmed the existence of *bcc* CuZn alloy in most of our samples, especially those with higher Zn^0^ concentration, i.e. CZG29 gives highest quantity of *bcc* CuZn alloy.

### DFT Modeling

**Figure 4:**
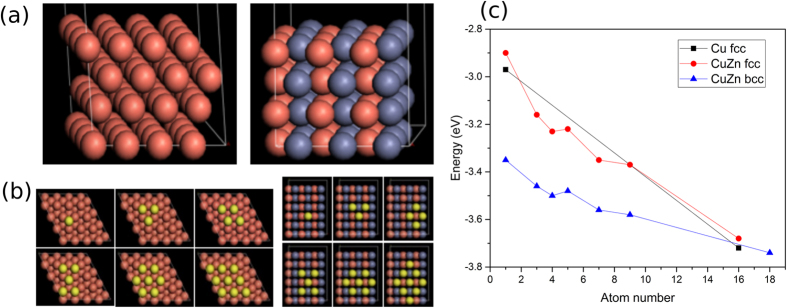
(**a**) Cu *fcc* (111) 4 × 4 model and 50%Cu50%Zn *bcc* (110) 3 × 3 model; (**b**) additional 1, 3, 4, 5, 7 and 9 Cu atoms (yellow) on Cu *fcc* and 50%Cu50%Zn *bcc* surfaces; (**c**) plot of energy change of *fcc* Cu, *fcc* CuZn and *bcc* CuZn surfaces when adding increased number of Cu atoms onto those surfaces.

In the present work, *fcc* Cu (or *fcc* CuZn) and *bcc* CuZn can be clearly observed after the reduction procedure. It is intriguing to find that the sample containing a higher quantity of *bcc* CuZn shows better stability against particle sintering when high temperature treatment is applied. It has recently been reported that SiO_2_ supported Cu nanoparticles continue to grow, with an Ostwald ripening process, during DMO hydrogenation, causing deactivation of the Cu catalysts^35^. To further elucidate the Zn stabilization mechanism, DFT calculations were introduced. Fig. 4a illustrates the Cu *fcc* (111) 4 × 4 model (the similar surface *fcc* 50%Cu50%Zn (111), not shown) and 50%Cu50%Zn *bcc* (110) 3 × 3 model we adopted for the calculations. Additional Cu atoms (yellow) are placed on their surfaces progressively, as shown in Fig. 4b. As shown in Fig. 4c, the energy changes with increasing numbers of Cu atoms added to the surfaces containing exclusively topmost copper atoms (6 layered- *fcc* (111) Cu, *fcc* 50%Cu50%Zn (111) alloy and *bcc* 50%Cu50%Zn (110), Fig. S9). This simple calculation was used to model the reverse Ostwald ripening process; the higher stabilization energy for Cu atoms over this *bcc* CuZn surface reduces the Cu atoms migration, hence decreases the sintering extent. It is interesting to note, from Fig. 4c, that both *fcc* Cu and *fcc* CuZn show similar energy stabilizations upon addition of Cu atoms despite their difference in chemical composition. It can be clearly seen that the *bcc* CuZn surface, with the same Zn content as *fcc* CuZn, gives the highest energy stabilization to accommodate additional Cu atoms as compared to the other two surfaces. This clearly suggests that Cu atoms on *bcc* CuZn surface are more stable than the corresponding *fcc* Cu or *fcc* CuZn surfaces, which reduces the extent of sintering of Cu atoms due to the stronger geometric effects holding these atoms in position. Previous theoretical studies suggested that the phase change from *fcc* to *bcc* structure in CuZn when Zn ≥ 50% is attributed to the electronic energy stabilization of the system^34^. It is anticipated that this *bcc* arrangement can also exert stronger binding to Cu adatoms than that of *fcc*. This information also matches with known stronger binding properties of *beta* brass (*bcc* CuZn) in metallurgy, which can only be worked with hot, are harder, stronger and more suitable for casting. In contrast, *alpha* brass (*fcc* CuZn) is more malleable with weaker binding. It can be worked with cold and is used in pressing, forging or similar applications^36^. In this case, the dispersion of Cu atoms in nanosize *bcc* CuZn structure is shown to be more stable and can be used in Cu catalysts maintaining small CuZn clusters so as to keep their superior catalytic performance.

### Concluding remarks

Nano alloys play a crucial role in many heterogeneous catalytic processes and their applications are expected to rise rapidly. This is due to the vast number of configurations and type of surface sites that multi-component materials can present. However, characterization of small alloy clusters of different compositions and temperature pre-treatments with respect to catalytic performance is technically challenging. In this case, using a range of surface sensitive, diffraction and microscopic techniques, we report for the first time that a clear structural role of Zn inclusion into the Cu nano-clusters by forming stable nano-beta phase CuZn. The structure can offer a good dispersion of active surface Cu atoms for catalysis and more importantly, stabilize them from aggregation. Regarding the hydrogenation of DMO the key challenge is to identify a new method to stabilize the Cu surface. A method to maintain higher Cu dispersion and thermal stability against sintering would provide an excellent direction to improve catalytic performance. We show that using highly dispersed *bcc* Cu-Zn catalysts may provide a good starting point for improving this reaction.

## Methods

### Synthesis of CuZnGa catalysts

CuZnGa catalysts were synthesized using a pH-controlled co-precipitation method. The metal precursors were hydrated metal nitrate salts: Cu(NO_3_)_2_·3 H_2_O (Aldrich), Zn(NO_3_)_2_·6 H_2_O (Aldrich), and Ga(NO_3_)_3_·9 H_2_O (Aldrich). For a typical preparation, the metal nitrates [3.77 g Cu(NO_3_)_2_·3 H_2_O; 5.53 g Zn(NO_3_)_2_·6 H_2_O; 0.75 g Ga(NO_3_)_3_·9 H_2_O] were dissolved completely in 100 mL deionized water. A Na_2_CO_3_ aqueous solution was prepared by dissolving 3.50 g of Na_2_CO_3_ in 100 mL of DI water. The solutions were added simultaneously into a plastic reactor containing 250 mL of preheated DI water. A delivery pump with two 50 mL syringes was used to inject the precursor metal nitrate solution at a constant rate of 0.42 mL/min in an automatic and reproducible manner. An HPLC pump was used to deliver the Na_2_CO_3_ solution at a rate of 0.35–0.70 mL/min. The mixture was stirred at 1000 rpm, with pH of the precipitating solution carefully maintained at 6.5. The precipitation process took place at around 80 °C. The pH of the liquid was measured using a temperature-dependent pH meter and was controlled at pH 6.5, with an error range of ± 0.1. Once the addition of the precursor metal nitrate solution was completed, the first aging was carried out under atmospheric conditions to let the pH value to become stable. After 30 min, the pH was measured again to ensure that the target pH had been reached before putting the lid onto the reactor. The resulting precipitate was continued to the second aging process in solution at 100 °C for 15 h. After aging, the precipitate was extracted by centrifugation at 5000 rpm. The centrifuged precipitate was washed with DI water five times at 5000 rpm to remove residual Na^+^ ions. The resulting wet solid was dried in air at 80 °C overnight and then calcined in static air, at a ramp of 5 °C/min up to desired temperature (330 °C, if not indicated) for 3 h to produce the final catalyst. For the Cu/SiO_2_ catalyst, the synthesis details could be found in the literature^12^.

### DMO hydrogenation reaction

Catalytic reactions were conducted using continuous flow in a stainless steel tubular reactor equipped with a computer-controlled auto-sampling system. 100 mg of as-calcined catalyst with the particle diameter of 0.25 mm to 0.42 mm (40–60 meshes) was placed in the center of the reactor, and both sides of the catalyst bed were packed with quartz powders (40–60 meshes). Prior to the evaluation of the catalytic performance, the catalyst precursor was pre-reduced in a 5%H_2_-95%N_2_ atmosphere at 623 K for 4 h at a ramping rate of 2 °C/min. The catalyst bed was then cooled to the reaction temperature. Pure H_2_ was fed into the reactor and the system pressure was held at 3.0 MPa with the aid of a back-pressure regulator. For the hydrogenation of DMO, a 12 wt.% DMO-methanol solution was pumped into the catalyst bed with varying weight liquid hourly space velocity (WLHSV_DMO_) using a Series III digital HPLC pump (Scientific Systems, Inc.). Under a given condition, the outlet stream was sampled using an automatic Valco six-port sampling valve at 30 min intervals after the reaction reached a steady state. The products were analyzed using an online gas chromatograph (Shimadzu GC-2010) equipped with a DB-Wax capillary column (30 m × 0.32 mm × 0.25 μm) and a flame ionization detector with a relative standard deviation (RSD) of less than 2%. The RSD of analysis data for each sampling was less than 3%. The products were also collected and confirmed using a 7890GC–5975 MS system.

## Additional Information

**How to cite this article**: Li, M. M.-J. *et al.* The remarkable activity and stability of a highly dispersive *beta*-brass Cu-Zn catalyst for the production of ethylene glycol. *Sci. Rep.* **6**, 20527; doi: 10.1038/srep20527 (2016).

## Supplementary Material

Supporting Information

## Figures and Tables

**Table 1:** XPS of CuZnGa samples with variations in composition and calcination temperature.

Sample	Composition Cu:Zn:Ga	Zn/Ga	Zn/Cu	Zn^0^/ (Zn^0^ + Zn^2+^)	Zn^0^/Ga	Zn^0^/Cu
CZG34	43:56.5:0.5	87.68	3.66	0.19	16.66	0.70
CZG33	43:54.5:2.5	18.03	3.47	0.22	3.97	0.76
CZG29	43:52:5	7.1	4.14	0.32	2.72	1.32
CZG28–280	43:47:10	3.05	1.93	0.29	0.92	0.58
CZG28–330	43:47:10	5.97	3.39	0.30	1.79	1.02
CZG28–380	43:47:10	4.71	3.61	0.20	0.94	0.72
CZG28–450	43:47:10	4.72	2.99	0.18	0.85	0.54
CZG28–500	43:47:10	4.06	2.04	0.17	0.69	0.35
CZG30	43:37:20	2.03	4.01	0.2	0.53	0.8
CZG31	43:28.5:28.5	0.97	2.1	0.14	0.14	0.29
CZG32	43:19:38	0.43	2.4	0.1	0.08	0.24

**Table 2:** EXAFS of CuZnGa samples of various calcination temperatures and chemical compositions.

Sample	Cu:Zn:Ga	Enot*	CN (*bcc* Cu-Zn & *fcc* Cu-Cu)	(Cu-Zn & *fcc* Cu-Cu)	Bond length (Å) (Cu-Zn & *fcc* Cu-Cu)	CN (*bcc* Cu-Cu)	DW-factor (*bcc* Cu-Cu)	Bond length (Å) (*bcc* Cu-Zu)	R-factor
CZG28–280	43:47:10	4.2	6.9 (4)	0.012 (1)	2.55 (1)	1.1 (4)	0.013 (5)	2.96 (3)	0.8%
CZG28–330	43:47:10	4.8	6.4 (3)	0.010 (1)	2.53 (1)	1.5 (5)	0.014 (4)	2.95 (3)	0.6%
CZG28–380	43:47:10	4.7	7.6 (4)	0.011 (1)	2.54 (1)	1.0 (5)	0.014 (5)	2.98 (4)	0.8%
CZG28–450	43:47:10	4.2	6.4 (3)	0.010 (1)	2.54 (1)	0.6 (3)	0.011 (5)	3.03 (4)	0.7%
CZG28–500	43:47:10	4.5	9.3 (3)	0.010 (1)	2.54 (1)	None	None	None	0.4%
CZG29	43:52:5	0.5	7.5 (4)	0.010 (1)	2.54 (1)	1.8 (6)	0.014 (3)	2.94 (1)	0.5%
CZG30	43:37:20	1.5	7.3 (2)	0.008 (1)	2.54 (1)	1.0 (4)	0.013 (4)	3.00 (3)	0.4%
CZG32	43:19:38	3.1	8.2 (2)	0.009 (1)	2.54 (1)	0.6 (1)	0.003 (2)	3.01 (2)	0.4%

^*^Enot is the energy difference of absorption energy in experimental value and calculated value.
