# Supplementary material for: The remarkable activity and stability of a highly dispersive beta-brass Cu-Zn catalyst for the production of ethylene glycol
Source: Sci Rep. 2016 Feb 9;6:20527. doi: 10.1038/srep20527 (PMC4746617; doi:10.1038/srep20527)
Supplement: Supporting Information [file srep20527-s1.pdf]

The remarkable activity and stability of a highly dispersive *beta*-brass Cu-Zn catalyst for the production of ethylene glycol

Molly Meng-Jung Li[1], Jianwei Zheng[2], Jin Qu[1], Fenglin Liao[1], Elizabeth Raine[1], Winson C. H. Kuo[1], Shei Sia Su[3], Pang Po[3],Youzhu Yuan[2*], Shik Chi Edman Tsang[1*]

[1] *Wolfson Catalysis Centre, Department of Chemistry, University of Oxford, Oxford, OX1 3QR, UK*

[2] *State Key Laboratory of Physical Chemistry of Solid Surfaces and National Engineering Laboratory for Green Chemical Production of Alcohols-Ethers-Esters, Collaborative Innovation Center of Chemistry for Energy Materials, College of Chemistry and Chemical Engineering, Xiamen University, Xiamen 361005, PR China*

[3] *Metallurgy and Materials, University of Birmingham, B15 2TT, UK*

*Correspondence to: edman.tsang@chem.ox.ac.uk

Supplementary Information

Powdered x-ray diffraction (PXRD)

The X-ray diffraction (XRD) profile was collected by a Philips PW-1729 diffractometer with Bragg-Brentano focusing geometry using Cu Ka radiation (lambda= 1.5418 Å) from a generator operating at 40 kV and 40 mA. Table S1 shows the Phase symbol, chemical formula and PDF number which are used in this work.

Table S1. Phase symbol, chemical formula and PDF number which are used in this work.

Phase symbol	Formula	PDF#
A: aurichalcite	$(Cu,Zn)_5(CO_3)_2(OH)_{16}$	82-1253
Z: zincite	ZnO	36-1451
T: tenorite	CuO	05-0661
S: Spinel structure	$ZnGa_2O_4$	86-0415
#: Aluminum	Al	85-1327

Transmission electron microscopy (TEM)

High-resolution TEM (HRTEM) measurements were performed on a Tecnai F30 electron microscope (Phillips Analytical) operated at an acceleration voltage of 300 kV.
Specimens were crushed and dusted onto a holey carbon coated Cu grid. In addition, field emission TEM/STEM (FEI Technai F20S-TWIN) was conducted at Johnson Matthey using EDAX Si(Li) LN2 EDS detector with SUTW (super ultra-thin window) of active area of 30mm^2 at the energy resolution of 135eV measured at Mn K alpha.

Temperature programmed and reduction (TPR)

Temperature-programmed reduction (TPR) measurements were obtained using a ThermoQuest TPRO 110 instrument. Inside the TPR quartz tube, 0.026 g of the calcined catalyst sample was sandwiched between two layers of glass wool with a thermocouple placed in contact with the sample. The TPR tube was then inserted into the instrument for a helium pretreatment. The helium gas pretreatment (He running through the TPR tube at 10 mL/min at a temperature ramp of 10 °C/min from 40 to 150 °C, then held for 5 min before cooling) cleaned the catalyst surface by removing any absorbed ambient gas molecules. After the pretreatment, a reduction treatment (5% H_2 in Argon flowing through the TPR tube at 20 mL/min at a temperature ramp of 10 °C/min from 40 to 330 °C, then held at 330 °C for 30 min before cooling to room temperature) was carried out to reduce the Cu^{2+} within the sample. Cu(II)O was reduced to Cu^0 by the flow of hydrogen gas in the reduction treatment. The consumption of hydrogen gas changed the conductivity of the gas stream; hence, the change in conductivity was measured and calibrated as a function of both temperature and time to produce the TPR profile.

X-ray photoelectron spectroscopy (XPS)

XPS was performed using a Quantum 2000 Scanning ESCA Microprob instrument (Physical Electronics) equipped with an Al Kα X-ray radiation source (hv = 1486.6 eV). A flood gun with variable electron voltage (from 6 eV to 8 eV) was used for charge compensation. The raw data were corrected for substrate charging with the BE of the C peak (284.5 eV), as shown in the XPS handbook. The measured spectra were fitted using a least-squares procedure to a product of Gaussian–Lorentzian functions after removing the background noise. The concentration of each element was calculated from the area of the corresponding peak and calibrated with the sensitivity factor of Wagner.

High-sensitivity low-energy ion scattering (HS-LEIS)

HS-LEIS measurements were carried out on an IonTOF Qtac100 low-energy ion scattering analyzer. Ne^+ ions with a kinetic energy of 5 keV were applied at a low ion flux equal to 445 pA cm^{-2}. The surface composition was obtained from the area of the corresponding peak and calibrated with the sensitivity factors.

(a) (b)

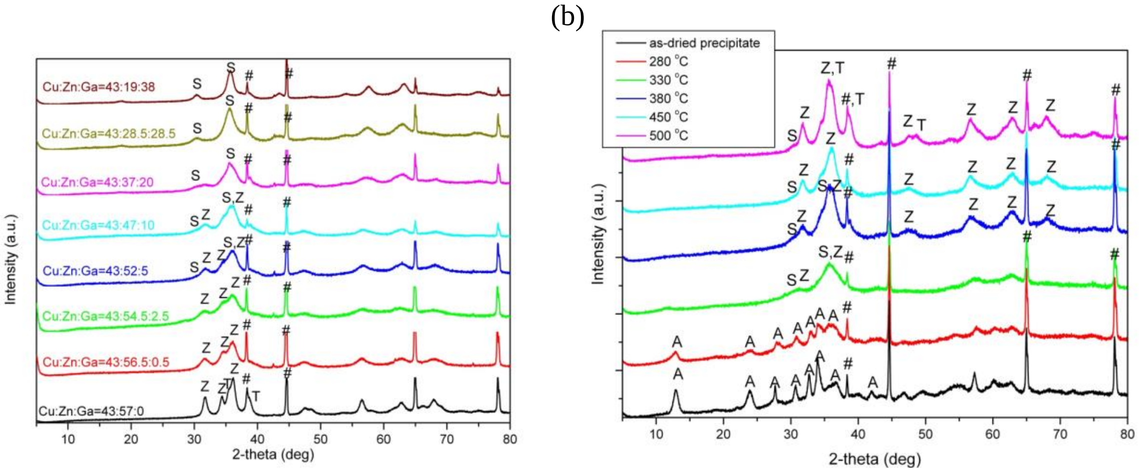

Figure S1. XRD profiles of calcined CuZnGa catalyst with various (a) chemical compositions; (b) calcination temperatures.

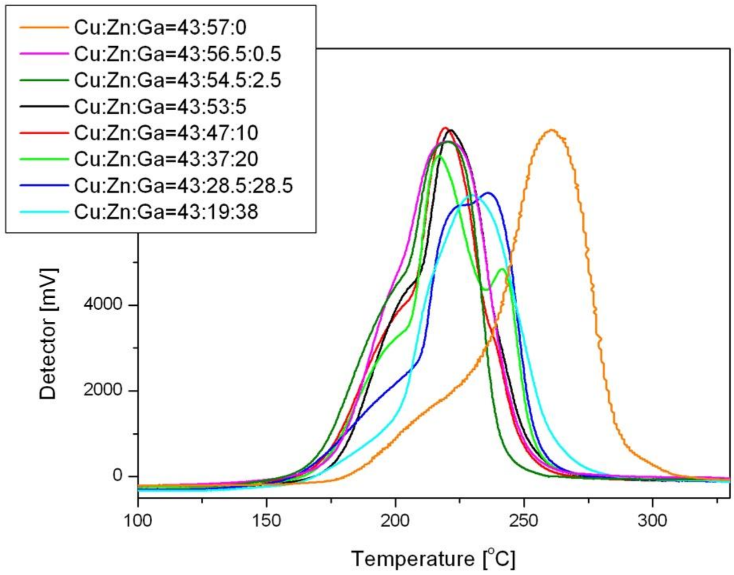

Figure S2. TPR profiles of calcined CuZnGa catalyst with different chemical compositions.

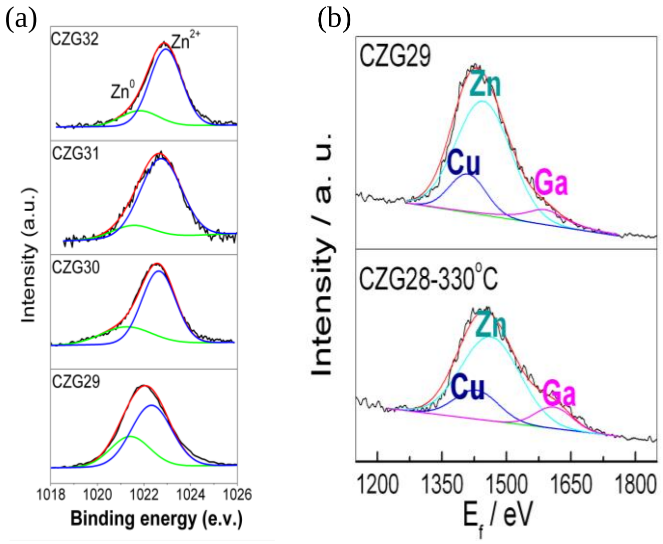

Catalyst	Method	Zn/Cu Atomic ratio	Zn/Ga atomic ratio
CZG29	HS-LEIS	4.42	7.89
	XPS	4.14	7.1
CZG28-330oC	HS-LEIS	3.87	6.29
	XPS	3.39	5.97

Figure S3. (a) XPS Zn 2p peaks of CZG29, CZG30, CZG31 and CZG32 samples; (b) HS- LEIS spectra at 5 keV Ne^+ and the insert table showing the comparison of Zn/Cu and Zn/Ga ratios obtained from XPS and HS-LEIS for CZG29 and CZG28-330°C samples.

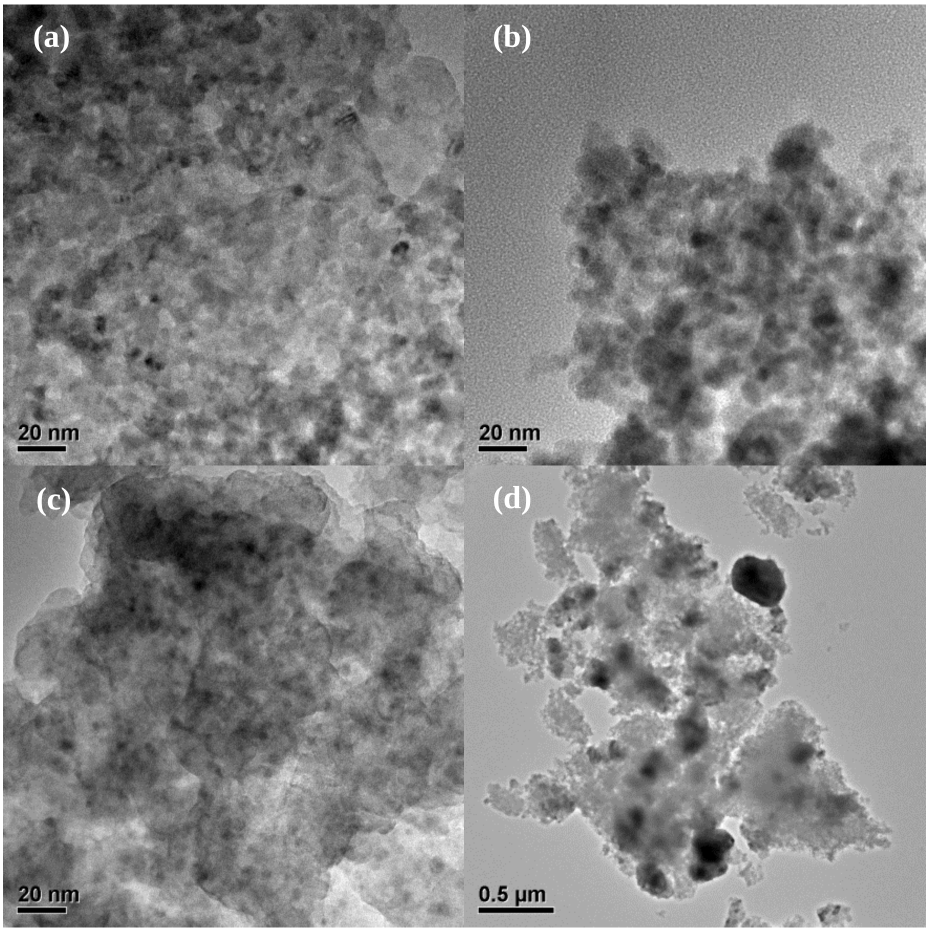

Figure S4. TEM images of before (a) and after (b) 24h heat treatment of CZG29; before (c) and after (d) 24h heat treatment of Cu/SiO_2

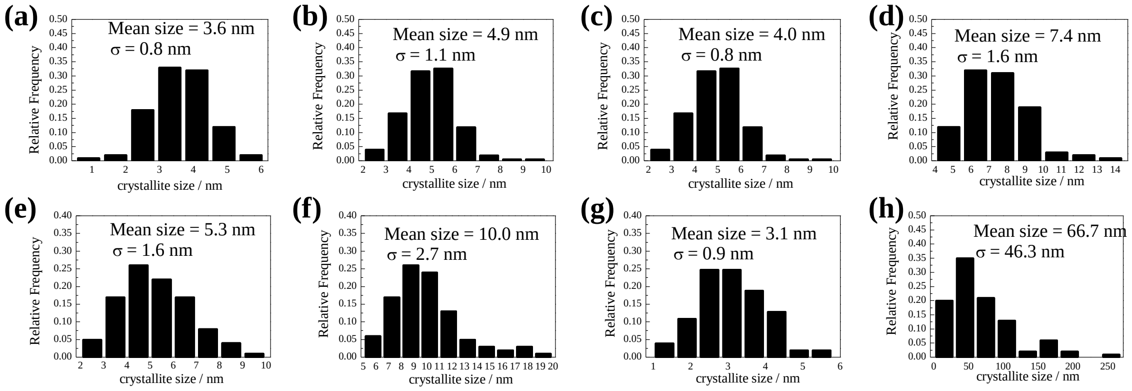

Figure S5. (a) Reduced CZG-29; (b) Used CZG-29; (c) Reduced CZG28-380 °C; (d) Used CZG28-380 °C; (e) Reduced CZG28-450 °C; (f) Used CZG28-450 °C; (g) Reduced 40%Cu/SiO_2; (h) Used 40%Cu/SiO_2

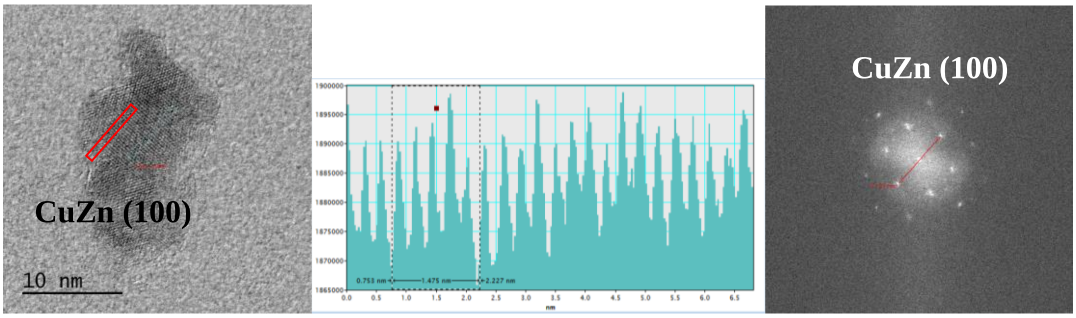

Figure S6. d-spacings analyses from lattice fringes and corresponding fast-Fourier transform (FFT) images of selected HR-STEM area of CZG29 sample which contains crystallite region of *bcc* CuZn (100).

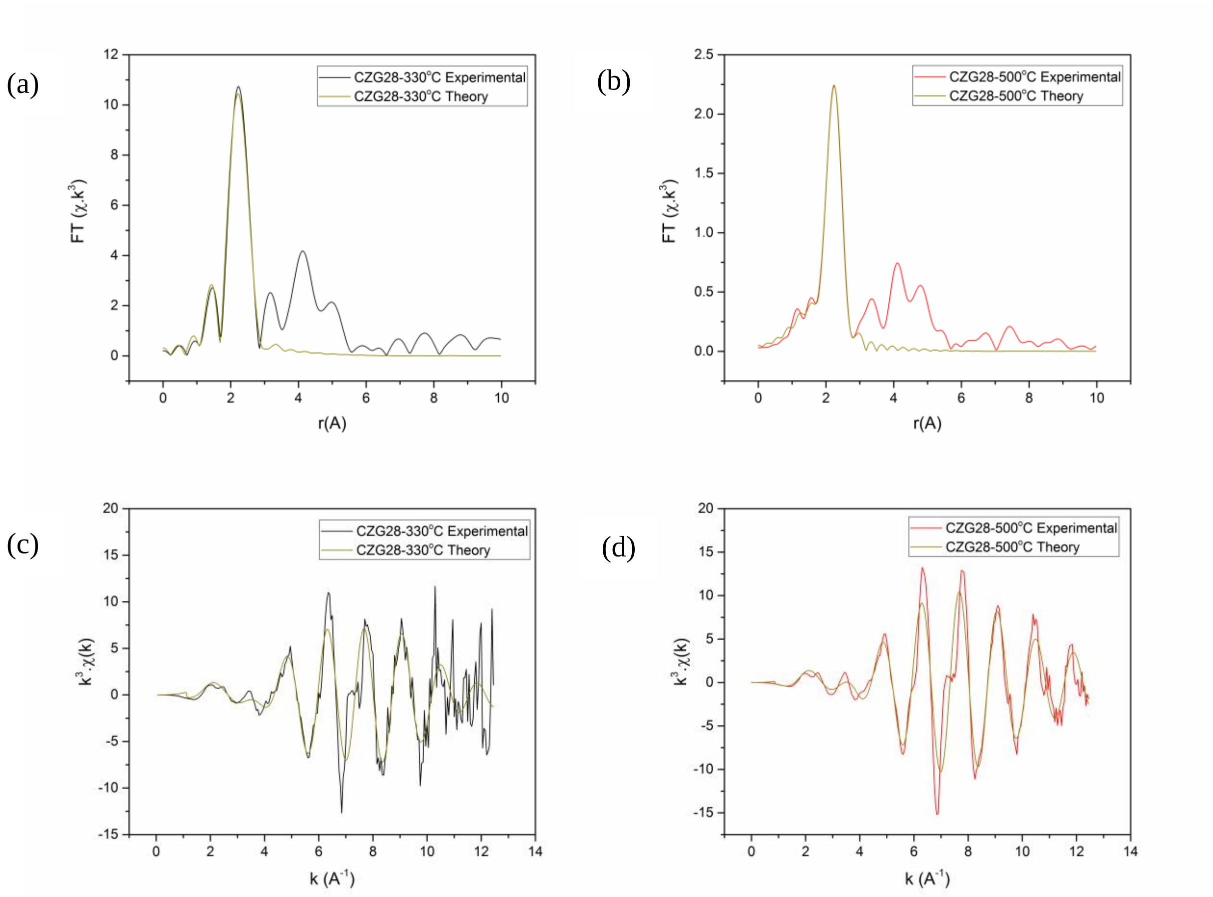

Figure S7. EXAFS plots of: (a) $k^3.\chi$ phase corrected Fourier transform of experimental and fitted data for CZG28-330 ^oC; (b) $k^3.\chi$ phase corrected Fourier transform of experimental and fitted data for CZG28-500 ^oC; (c) $k^3.\chi$ experimental and fitted data for CZG28-330 ^oC; (d) $k^3.\chi$ experimental and fitted data for CZG28-500 ^oC. The heights of the first shell contributions have been equalised.

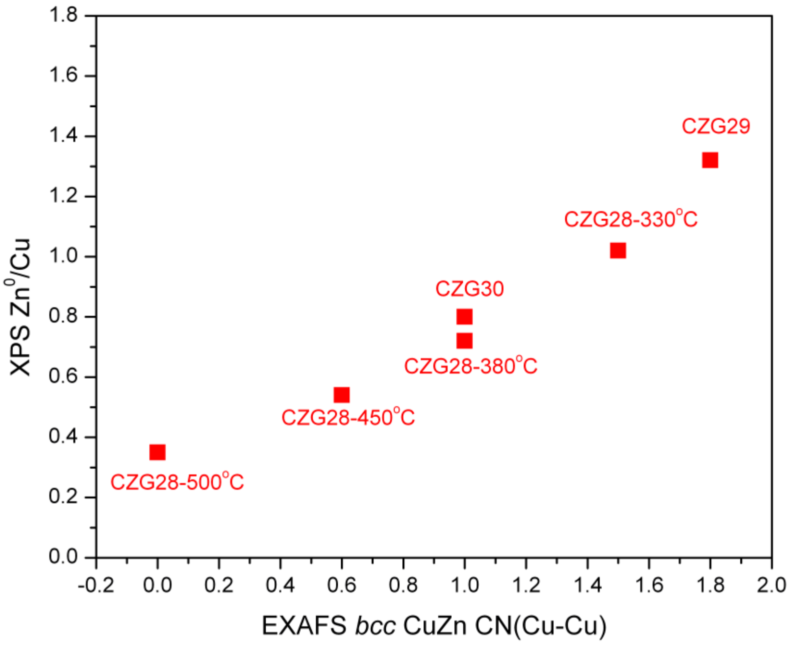

Figure S8. The correlation between Zn^0/Cu obtained from XPS result with *bcc* CuZn coordination number obtained from EXAFS.

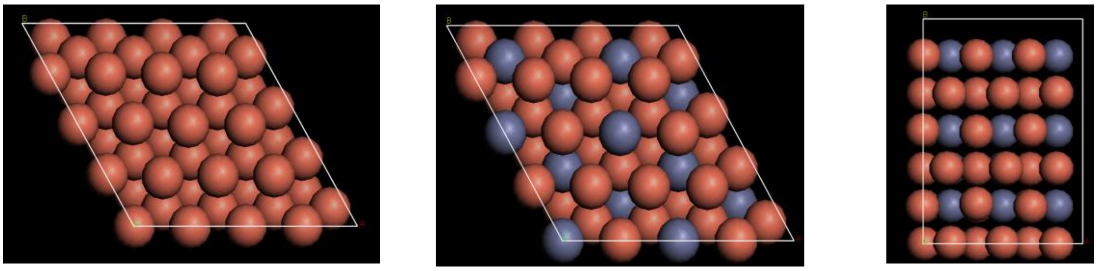

Figure S9. The surface sites that host additional Cu atoms on top of 6-layered *fcc* Cu; *fcc* 50%Cu50%Zn and *bcc* 50%Cu50%Zn. These surfaces were selected for DFT calculations due to absence of exposed Zn atom on the topmost layer hence localized electronic factor is minimized.
